# Human Paraoxonase-1 Activity Is Related to the Number of CD4+ T-Cells and Is Restored by Antiretroviral Therapy in HIV-1-Infected Individuals

**DOI:** 10.1155/2014/480201

**Published:** 2014-02-27

**Authors:** Luciana Morganti Ferreira Maselli, Joel da Cunha, Eliana Battaggia Gutierrez, Raul Cavalcante Maranhão, Celso Spada, Sérgio Paulo Bydlowski

**Affiliations:** ^1^Laboratory of Genetics and Molecular Hematology (LIM31), University of São Paulo School of Medicine, 05403-000 São Paulo, SP, Brazil; ^2^Research Division, Pró-Sangue Hemocentro de São Paulo Foundation, 05403-000 São Paulo, SP, Brazil; ^3^Department of Clinical Laboratory, Health Sciences Center, Federal University of Santa Catarina, 88040-900 Florianópolis, SC, Brazil; ^4^Clinics of Parasitic and Infectious Diseases, Hospital and Clinics of the University of São Paulo School of Medicine, 05403-000 São Paulo, SP, Brazil; ^5^Lipid Metabolism Laboratory, Heart Institute, University of São Paulo School of Medicine, 05403-000 São Paulo, SP, Brazil

## Abstract

*Background*. Paraoxonase-1 (PON1) activity is suggested to be altered in individuals infected with human immunodeficiency virus type-1 (HIV-1). We investigated PON1 activity in individuals receiving different regimens of highly active antiretroviral therapy (HAART). *Methods*. PON1 activity was evaluated in 91 HIV-1 seronegative and 624 HIV-1 infected individuals (115 were not undergoing therapy (ART-naïve), and 509 were receiving HAART). HIV-1 infected individuals were treated with the following: efavirenz (EFV; *n* = 195) or nevirapine (NVP; *n* = 95) or lopinavir/ritonavir (LOP/r; *n* = 219). Serum levels of total cholesterol (TC), HDL, and low-density lipoprotein (LDL) fractions and the atherogenic indices (AI, TC : HDL, and LDL : HDL ratios) were determined. *Results*. PON1 activity (U/L) was lower in the ART-naïve group compared with the other groups. PON1 activity correlated with CD4+ T-cell number of ART-naïve group (*r* = 0,121; *P* = 0,014). The LOP/r group showed a reduction in HDL and an increase in AI (TC : HDL ratio) in comparison with other groups. *Conclusion*. PON1 activity was reduced in untreated individuals, but not in individuals receiving HAART. PON1 activity correlated with the number of CD4+ T-cells. The findings suggest that the activity of PON1 is associated with the immune status of HIV-1 infected individuals.

## 1. Introduction

Changes in lipid metabolism are frequently found in individuals infected with human immunodeficiency virus type-1 (HIV-1) [1, 2]. The dyslipidemia has been associated with an increased cardiovascular risk in individuals with HIV-1 and can be exacerbated by the use of highly active antiretroviral therapy (HAART), especially in individuals treated with protease inhibitors (PI) [3, 4]. The use of PI currently constitutes the most potent option against HIV-1, preventing the maturation of viral particles and effectively controlling the infection of new cells by the virus [5]. The common mechanisms by which PI promote these changes remain unknown. However, the main effect of PI appears to be the suppression of the breakdown of the nuclear form of sterol-regulatory element binding protein-1 (nSREBP1) in the liver and adipose tissue. This regulator is a key element in the proteolytic pathway responsible for regulating cellular and plasma levels of fat and cholesterol [6]. In this context, HAART and HIV-1 infection *per se* may represent a greater risk for cardiovascular and lipid disorders. The latter is characterized by changes that may include high concentrations of total cholesterol (TC), triglycerides (TG), and low-density lipoprotein (LDL) cholesterol and a significant reduction in the high-density lipoprotein (HDL) cholesterol fraction, which may be accompanied by a change in the enzymatic activity of paraoxonase-1 (PON1) [6, 7]. PON1 is an antioxidant enzyme present in serum that is strongly associated with apolipoprotein-A1 (apoAl) from HDL and protects LDL against oxidative modifications. The action of serum PON1 most likely occurs through the involvement of the enzyme in reverse cholesterol transport, a well-established antiatherogenic property of HDL [8, 9]. PON1 has the ability to inhibit LDL oxidation (oxLDL) and significantly reduce the lipid peroxidase enzyme, which decreases the accumulation of cholesterol in peripheral tissues [9]. The oxidative modification of LDL in the arterial wall plays a central role in the pathogenesis of atherosclerosis, which is characterized by the deposition of lipids and the formation of atherosclerotic plaques that cause narrowing of the blood vessels [10]. The inhibition of LDL oxidation by HDL is attributed to the high antioxidant content of this lipoprotein due to the antioxidant properties of apoA1 and by the presence of other different antioxidant enzymes, such as glutathione peroxidase and PON itself, which prevent the formation of or degrade bioactive products of LDL oxidation [11, 12]. Some studies have shown that the activity of PON1 may be affected and/or inactivated by oxidative stress, which could explain its reduced activity during HIV-1 infection [7, 13]. In individuals infected with HIV-1 without HAART and/or with acquired immunodeficiency syndrome (AIDS), there is an increase in oxidative stress characterized by increased plasma metabolites of lipid peroxidation and/or a quantitative decrease in antioxidants compared to seronegative controls considered to be in a healthy condition [14, 15]. Additionally, it seems that this reduction is associated with a decrease in the number of CD4+ T-cells and indicates that reduced PON1 activity is affected by the immune status of these individuals. Baseline CD4+ T-cell counts remain the most relevant predictors of clinical progression and survival in HIV-1-infected individuals receiving HAART [15, 16]. Therefore, possible reductions in the activity of PON1 and HDL concentrations may characterize an increased cardiovascular risk in individuals infected with HIV-1, while the quantification of CD4+ T-cells may predict the immune status of those individuals regardless of whether they are receiving HAART. Based on this hypothesis, we aimed to evaluate the existence of a correlation between serum PON1 activity and the number of CD4+ T-cells and determine the impact of different HAART regimens on PON1 and lipid metabolism.

## 2. Materials and Methods

### 2.1. Individuals and Study Design

The study was conducted from April 2007 to November 2012 on 715 volunteers (age ≥18 years). Of these, 624 HIV-1-infected were treated at the Casa da AIDS of HCFMUSP, the University Hospital of the Federal University of Santa Catarina (HU/UFSC), and the Clinical Hospital University of São Paulo Medical School (HCFMUSP). HIV-1 seronegative individuals (*n* = 91) from the blood bank at HU/UFSC were also enrolled in the study (Table 1). In the group of HIV-1-infected individuals, 115 were not undergoing the HAART regimen (ART-naïve), 290 were using a HAART regimen consisting of a nonnucleoside reverse transcriptase inhibitor (NNRTI-based) with 600 mg efavirenz (EFV) once daily (*n* = 195) or 200 mg nevirapine (NVP) twice daily (*n* = 95) in association with a nucleoside reverse transcriptase inhibitor (NRTI), and 219 were using 400 mg/100 mg lopinavir/ritonavir (LOP/r) once daily (PI-based) in association with an NRTI (Table 2). The volunteers were informed of the need for a single blood collection, and all subjects included in this study signed an informed consent form according to the protocol approved by the Ethics Committee on Human Research of HU/UFSC (CEPSH: 289/09) and HCFMUSP (CAPPesq: 539/04 and 1185/09). Individuals were excluded from the study if they were diagnosed with hepatitis or if they had used hypoglycemic agents, hypolipidemic agents, antioxidants, anti-inflammatory steroids, or steroids in the three months before the day that the biological material was obtained on. The group was strictly followed up, both clinically and in the laboratory, every ninety days, and no opportunistic infections were observed. Demographic and anthropometric data are shown in Table 1.

### 2.2. Blood Sample Collection

Blood was obtained by antecubital venous puncture using a vacuum system (Vacutainer, Becton, Dickinson Co., New Jersey, USA) in the morning after subjects were fasted for 12 to 14 hours. Serum samples were obtained by centrifuging the blood in a CELM model LS-II centrifuge at 2,500 rpm (1,050 ×g) at 4°C for 10 minutes. The samples were then divided into 300 *μ*L aliquots, transferred to cryogenic tubes, and stored in liquid nitrogen at −180°C until use.

### 2.3. Determination of PON1 Activity

Basal PON1 (U/L) levels were determined according to the method of Sentí and colleagues (2003) [17] employing paraoxon (O, O-diethyl O-paranitrophenyl phosphate) as a substrate (Sigma-Aldrich Chemical Co., Saint Louis, USA). The enzymatic hydrolysis of paraoxon releases *p*-nitrophenol, whose formation rate was determined by spectrophotometry. The assay was performed in triplicate for each sample. An aliquot of 25 *μ*L of serum from each patient was thawed at room temperature and diluted with 500 *μ*L of buffer (0.1 M Tris-HCl, pH = 8.0, 2 mg/dL CaCl_2_, and 5.5 mmol paraoxon/L), and 320 *μ*L was transferred into 96-well flat bottom microplates. The absorbance was then read every minute for 10 minutes with a microplate reader (Microplate Reader, Benchmark, Bio-Rad, Hercules, CA, USA) at a wavelength of 405 nm at 37°C.

### 2.4. Serum Lipids and Atherogenic Indices

Serum levels of TC, TG, and HDL were determined enzymatically (Dade Behring Inc., Newark, USA). Serum levels of LDL were determined using the Friedewald formula [18]. Atherogenic indices (AI) were calculated using the relationships between the parameters to obtain TC : HDL and LDL : HDL ratios [19, 20].

### 2.5. Markers of Infection

HIV-1 RNA was quantified using plasma and the commercially available Nucleic Acid Sequence Based Amplification kit (NASBA, Organon Teknika, Boxtel, the Netherlands). The minimum detection limit indicated by the manufacturer is 50 copies/mL of HIV-1 RNA. Subpopulations of CD4+ T-cells, CD8+ T-cells, and CD3+ T-cells and CD4+ : CD3+ and CD8+ : CD3+ cells were determined by three-color flow cytometry using monoclonal antibodies and a Becton/Dickinson FACScount flow cytometer (Becton/Dickinson, San Jose, CA, USA).

### 2.6. Statistical Analysis

The results were expressed as the arithmetic means plus standard deviations (mean ± SD) and medians [interquartile range (IQR): 95% confidence interval (CI)]. For comparison, we also used Student's *t*-test and an analysis of univariate variance for multiple comparisons (ANOVA) followed by Tukey's HSD (honestly significant difference) test. Correlation analysis between parameters was performed using Pearson's test or Spearman's test. All descriptive and statistical analyses were performed using the Statistical Package for Social Sciences software (SPSS, Inc., Chicago, USA) 12.0 and SAS 8e. Charts were constructed using GraphPad Prism version 5.0 (GraphPad Software Inc., La Jolla, USA).

## 3. Results

Tables 2 and 3 describe the different HAART regimens used and the results of the laboratory tests for the study groups, respectively. In the studied population, the parameters of age, body mass index (BMI), and homogeneity showed no marked differences (Tables 1 and 3). HIV-1 seropositive individuals treated with NVP or EFV or LOP/r PON1 activity showed no significant difference in comparison with the seronegative group. However, the ART-naïve group had the lowest PON1 activity, which was characterized by a significantly lower median compared to all groups (Table 4). These results suggest that infection has a negative effect on PON1 activity in HIV-1-infected individuals who do not use HAART compared with subjects undergoing treatment with different HAART regimens.

PON1 activity showed a significant positive correlation with the number of CD4+ T-cells in the ART-naïve group (*r* = 0.121, *P* = 0.014), but not in relation to the EFV or NVP group (*r* = 0.253,  *P* = 0.159) and the LOP/r group (*r* = 0.261, *P* = 0.266). PON1 activity showed no significant correlation with serum levels of HDL in the ART-naïve (*r* = −0.150, *P* = 0.111), EFV or NVP (*r* = 0.015, *P* = 0.796), and LOP/r (*r* = 0.114, *P* = 0.090) groups. These results suggest that the activity of PON1 is less affected in individuals possessing a higher number of CD4+ T-cells and undetectable HIV-1 RNA levels.

Median serum HDL levels in HIV-1 seropositive groups were below the seronegative group median and lower than the reference value of ≥40 mg/dL (Table 4). The serum HDL levels in the LOP/r group were significantly lower than those observed in the EFV or NVP and seronegative groups. The ART-naïve group showed no significant difference compared to the EFV or NVP group. These results suggest that LOP/r therapy and HIV-1 infection *per se* affected the HDL levels.

Serum TC levels showed no significant difference between evaluated groups, with medians that were within the reference values for TC (<200 mg/dL) (Table 4). Therefore, HIV-1 infection and the different HAART regimens did not appear to affect serum levels of TC. Serum TG levels showed significantly different medians. Serum TG in the LOP/r group was significantly higher than the other groups, with a higher median than the reference value of <190 mg/dL (Table 4). The EFV or NVP group had a significantly higher median compared to the ART-naïve and seronegative groups (Table 4). The results suggest that therapy with LOP/r affects serum TG. Serum levels of LDL showed medians within the normal range of 100 to 129 mg/dL. The results show that different HAART regimens did not affect this parameter in HIV-1 infection.

Atherogenic risk (AI) characterized by the evaluation of TC : HDL and LDL : HDL ratios showed that the LOP/r group had the highest AIs among the groups (Table 4). The ART-naïve group showed significantly higher AIs compared to the seronegative group. The EFV or NVP group had significantly lower AIs than the LOP/r group (Table 4). The LDL : HDL ratio was significantly higher for the LOP/r group compared to the seronegative group but not compared to the ART-naïve and EFV or NVP groups. The ART-naïve and EFV or NVP groups showed a greater AI than the seronegative group (Table 4). These results suggest that therapy with LOP/r is a major AI.

Markers of infection by HIV-1, such as CD4+ T-cells, CD8+ T-cells, and HIV-1 RNA, showed satisfactory medians in the treated groups (Table 3). The median numbers of CD4+ T-cells in the treated groups were significantly different in the compared ART-naïve group. The median number of CD8+ T-cells was not significantly different. HIV-1 RNA was undetectable, <50 copies/mL, in 80% of subjects in the EFV or NVP group and 86% in the LOP/r group. The ART-naïve group had elevated levels of HIV-1 RNA in comparison with other groups (Table 3). These data showed that groups of HIV-1-infected individuals treated with HAART had a satisfactory immune status while they were participating in the study.

## 4. Discussion

In this study we demonstrated that serum PON1 activity was reduced in the ART-naïve group, and no correlation was observed between PON1 activity and serum levels of HDL for any group of HIV-1 seropositive individuals. PON1 activity showed a significant positive correlation with the number of CD4+ T-cells in the ART-naïve group, suggesting that immune status may directly affect the activity of PON1. Other studies have shown that enzyme levels are reduced in ART-naïve individuals who present a reduced number of CD4+ T-cells [10, 16]. Therefore, the existence of a relationship between the number of CD4+ T-cells and PON1 activity, which would be directly related to the best course of HIV-1 infection, may be suggested. Parra and colleagues (2007) [7] found a reduction in PON1 activity with an increase in its concentration in HIV-1-infected individuals that could be explained by the addition of a higher quantity of free radicals to PON1, by lower serum concentrations of HDL and apoA1, and by changes in the liver that would influence the serum concentration of PON1 [17, 18]. In this study, the individuals receiving HAART had HIV-1 RNA levels undetectable and the ART-naïve group presented reduced HIV-1 RNA levels. HIV-1 RNA levels and CD4+ T-cells numbers are indicators of the clinical stage of infection and are considered prognostic factors for the evaluation of the course of HIV-1 infection. It remains uncertain whether the replication of HIV-1 virus itself would be related to PON1 status, and our results corroborate the results obtained by other studies [7, 19–21]. The maintenance of CD4+ T-cells and CD8+ T-cells numbers characterizes the efficacy of HAART [22–24], which inhibits viral replication, slows the progression of immunodeficiency, and therefore restores immunity, increasing the quality of life of HIV-1-infected individuals. The ability of HAART to induce changes in lipid metabolism, especially for those who are receiving PI, may cause an increased risk for cardiovascular disease and atherosclerosis [4, 21, 25]. In our study, the seropositive groups evaluated showed no changes in their serum levels of TC and LDL, and serum levels of HDL were significantly lower in the LOP/r group. Unlike PI-based regimens, NNTRI-based therapy is associated with an increase in serum HDL levels and is considered a less atherogenic therapy [2], which was demonstrated in groups treated with EFV or NVP. HDL collaborates with the innate immune system once it exerts antioxidant actions as a result of its association with enzymes such as PON, lecithin cholesterol acyltransferase (LCAT), and selenoglutathione peroxidase (GPX) [26–28]. Pereira and colleagues (2006) [29] demonstrated that individuals using NNRTI-based therapy had better serum levels of HDL and a lower risk for atherosclerotic diseases. Abnormal serum TG levels may be found in advanced stages of HIV-1 infection and in individuals with a high degree of immunosuppression, thus characterizing a state of immune activation in ART-naïve individuals [30, 31]. In groups treated with EFV or NVP and LOP/r, elevated serum TG levels were observed. Wohl and colleagues (2008) [6] studied dyslipidemia in individuals infected by HIV-1 and demonstrated the occurrence of high TG levels with a reduction in serum levels of HDL compared with seronegative individuals [2, 31]. The trend of a specific PI to cause elevated TG levels is variable, and a HAART regimen incorporating LOP/r impacts lipid metabolism in a way that may cause a significant elevation in TG and reduced HDL [32]. In this study, all of the 219 (100%) subjects treated with PI were using the LOP/r combination, which could explain, at least in part, the changes observed in the lipid profile. These results influenced the TC : HDL and LDL : HDL ratios, which characterize the AI and are important parameters for determining the risk for cardiovascular diseases [33, 34]. In this study, the group treated with LOP/r had a significantly higher TC : HDL ratio, and the LDL : HDL ratio was significantly higher for all groups of HIV-1-infected individuals. Estrada and colleagues (2011) [35] observed that individuals who were seropositive for HIV-1 had a higher cardiovascular risk when the TC : HDL ratio was evaluated and that this risk was more evident in women. In a study involving 130 seropositive individuals with undetermined viral load Sankatsing and colleagues (2011) showed that PI-based therapy promoted an increased risk for cardiovascular disease compared to treatment with an NNRTI-based regimen. Moreover, in ART-naïve individuals who began HAART with EFV or NVP, the serum levels of HDL and apoA1 increased such that the subsequent introduction of a PI resulted in a reduction in both proteins in the serum [35, 36]. In this study, 95 HIV-1-infected individuals were using NVP, and 200 were using EFV. Therefore, the results described here and in other studies suggest the prevalence of alterations in lipid metabolism in individuals who are seropositive for HIV-1 who have been treated with PI, as well as changes in the activity of the PON1 enzyme that are associated with HIV-1 infection. It is important to reinforce the idea that the survival of individuals who are seropositive for HIV-1 is accompanied by a chronic infectious process and continuous treatments with side effects, exposing these individuals to a number of complex situations that are multifactorial and interrelated, such as inflammation, atherosclerosis, and dyslipidemia. Furthermore, lipids, particularly cholesterol, are raw materials that are essential to the virus to ensure important steps of its cycle, such as establishment and replication [22]. One limitation of the present study is that it is a cross-sectional study. Thus, although a longitudinal clinical trial was performed with these HIV-1 infected individuals, a temporal analysis was not possible to be done. However, this study established that the likely factor that determines the reduction of PON1 activity is the suppression of the immune system by HIV-1.

## 5. Conclusion

Our results showed in a significant number of patients that the reduction of PON1 activity was associated with the number of CD4+ T-cells in HIV-1-infected individuals, ART-naïve, suggesting that immune status could interfere with the antiatherogenic activity of the PON1. Additionally, it was shown that therapy with LOP/r resulted in a higher AI. Individuals that maintained CD4+ T-cells within normal ranges and undetectable HIV-1 RNA levels, as a result of HAART, showed a restoration of the immune system function and of PON1 activity, which could lead and conduce to a lower risk for cardiovascular disease.

## Figures and Tables

**Table 1:** Baseline characteristics of the study subjects.

Parameters^(1)^	Seronegative (*n* = 91)	HIV-1-infected individuals (*n* = 624)			*P* value^(2)^
		ART-naïve (*n* = 115)	EFV or NVP (*n* = 292)	LOP/r (*n* = 217)	
Gender (M/F)	52/39	73/42	161/131	121/96	—
Age (years)	35 (26, 45)	38 (32, 44)	42 (22, 58)	41 (18, 59)	ns
BMI (kg/m^2^)	24 (22, 25)	21 (17, 24)	24 (18, 30)	23 (18, 32)	ns

NVP: nevirapine; LOP/r: lopinavir/ritonavir; M: male; F: female; BMI: body mass index (weight/height^2^ (kg/m^2^)); ns: no significant difference.

^(1)^Median (interquartile range (IQR) 25%–75%; 95% confidence interval (CI)).

^(2)^ *P* value: comparison between groups by ANOVA and Tukey's honestly significant difference.

**Table 2:** Highly active antiretroviral therapy (HAART) regimens of the study groups.

Antiretroviral therapy, *n* (%)^(1)^	HAART (*n* = 509)	
	EFV or NVP 292 (57)	LOP/r 217 (43)
NNRTI-based		
Zidovudine (AZT) 300 mg + lamivudine (3TC) 150 mg (bid) + efavirenz (EFV) 600 mg (qd)	153 (53)	—
Stavudine (d4T) 40 mg + lamivudine (3TC) 150 mg (bid) + efavirenz (EFV) 600 mg (qd)	26 (9)	—
Zidovudine (AZT) 300 mg + lamivudine (3TC) 150 mg (bid) + nevirapine (NVP) 200 mg (bid)	54 (18)	—
Stavudine (d4T) 40 mg + lamivudine (3TC) 150 mg (bid) + nevirapine (NVP) 200 mg (bid)	24 (9)	—
Tenofovir (TDF) 300 mg + lamivudine (3TC) 150 mg (bid) + efavirenz (EFV) 600 mg (qd)	16 (5)	—
Tenofovir (TDF) 300 mg + lamivudine (3TC) 150 mg (bid) + nevirapine (NVP) 200 mg (bid)	19 (6)	—
PI-based		
Zidovudine (AZT) 300 mg + lamivudine (3TC) 150 mg (bid) + lopinavir/ritonavir (LOP/r) 400 mg/100 mg (bid)	—	100 (47)
Stavudine (d4T) 40 mg + lamivudine (3TC) 150 mg (bid) + lopinavir/ritonavir (LOP/r) 400 mg/100 mg (bid)	—	117 (53)

Note: NNRTI: nonnucleoside reverse transcriptase inhibitor; PI: protease inhibitor; bid: twice daily; qd: once daily; TDF: tenofovir; AZT: zidovudine; 3TC: lamivudine; EFV: efavirenz; d4T: stavudine; NVP: nevirapine; LOP/r: lopinavir/ritonavir.

^(1)^Therapy with oral administration.

**Table 3:** Markers of infection and HIV-1 RNA in HIV-1-infected individuals with highly active antiretroviral therapy (HAART) regimens and ART-naïve.

Parameters^(1)^	HIV-1-infected individuals (*n* = 624)			*P* value^(2)^
	ART-naïve (*n* = 115)	EFV or NVP (*n* = 292)	LOP/r (*n* = 217)	
CD4+ T-cell (cells/mm^3^)	404 (205, 443)*	483 (334, 683)	486 (370, 635)	<0.05
CD8+ T-cell (cells/mm^3^)	955 (643, 1,340)	859 (607, 1,119)	952 (743, 1,215)	ns
CD4 : CD8 ratio	0.4 (0.3, 0.6)	0.5 (0.3, 0.7)	0.5 (0.2, 0.6)	ns
HIV-1 RNA (copies/mL^(3)^)	89,266 (±81,572)*	14,121 (±16,224)	16,301 (±20,014)	<0.05

EFV: efavirenz; NVP: nevirapine; LOP/r: lopinavir/ritonavir; ns: no significant difference.

^(1)^Median (interquartile range (IQR) 25%–75%; 95% confidence interval (CI)).

^(2)^ *P* value: comparison between groups by ANOVA and Tukey's honestly significant difference.

^(3)^Mean ± standard deviation (m ± SD).

*P < 0.05 when comparing with other groups.

**Table 4:** Laboratorial parameters and PON1 activity in HIV-1-infected individuals with highly active antiretroviral therapy (HAART) regimens and ART-naïve.

Parameters^(1)^	Seronegative (*n* = 91)	HIV-1-infected individuals (*n* = 624)			*P* value^(2)^
		ART-naïve (*n* = 115)	EFV or NVP (*n* = 292)	LOP/r (*n* = 217)	
PON1 activity (U/L)	117 (68, 218)	81 (51, 137)*	109 (60, 159)	106 (56, 157)	<0.05
HDL (mg/dL)	42 (40, 56)*	36 (31, 48)	37 (31, 47)	31 (28, 43)	<0.05
Total cholesterol (mg/dL)	183 (162, 203)	167 (144, 191)	190 (168, 217)	187 (155, 214)	ns
Triglycerides (mg/dL)	95 (76, 129)	104 (83, 145)	136 (101, 221)	196 (115, 241)*	<0.05
LDL (mg/dL)	114 (96, 122)	97 (75, 140)	108 (85, 128)	106 (71, 129)	ns
TC : HDL ratio^(3)^	3.0 (3.0, 3.8)*	4.4 (3.6, 6.3)	3,8 (2.9, 3.9)	5.9 (3.2, 6.8)*	<0.05
LDL : HDL ratio^(3)^	1,4 (1.0, 2.2)*	2.6 (1.8, 3.2)	2.5 (2.0, 3.2)	2.9 (2.0, 3.7)	<0.05

Note: EFV: efavirenz; NVP: nevirapine; LOP/r: lopinavir/ritonavir; BMI: body mass index (weight/height^2^ (kg/m^2^)); PON1: paraoxonase-1;

HDL: high-density lipoprotein; LDL: low-density lipoprotein; ns: no significant difference.

^(1)^Median (interquartile range (IQR) 25%–75%; 95% confidence interval (CI)).

^(2)^ *P* value: comparison between groups by ANOVA and Tukey's honestly significant difference.

^(3)^Atherogenic indices.

*P < 0.05 when comparing with other groups.
